# Essentials and guidelines for clinical medical physics residency training programs: executive summary of AAPM Report Number 249

**DOI:** 10.1120/jacmp.v15i3.4763

**Published:** 2014-05-08

**Authors:** Joann I. Prisciandaro, Charles E. Willis, Jay W. Burmeister, Geoffrey D. Clarke, Rupak K. Das, Jacqueline Esthappan, Bruce J. Gerbi, Beth A. Harkness, James A. Patton, Donald J. Peck, Robert J. Pizzutiello, George A. Sandison, Sharon L. White, Brian D. Wichman, Geoffrey S. Ibbott, Stefan Both

**Affiliations:** ^1^ University of Michigan Ann Arbor MI; ^2^ The University of Texas MD Anderson Cancer Center Houston TX; ^3^ Wayne State University Detroit MI; ^4^ UT Health Sciences Center San Antonio TX; ^5^ University of Wisconsin Madison WI; ^6^ Washington University St. Louis MO; ^7^ University of Minnesota Minneapolis MN; ^8^ Henry Ford Hospital System Detroit MI; ^9^ Vanderbilt University Nashville TN; ^10^ Upstate Medical Physics Victor NY; ^11^ University of Washington Seattle WA; ^12^ University of Alabama Birmingham AL; ^13^ Texas Oncology Round Rock TX; ^14^ University of Pennsylvania Philadelphia PA USA

**Keywords:** medical physics, education, training

## Abstract

There is a clear need for established standards for medical physics residency training. The complexity of techniques in imaging, nuclear medicine, and radiation oncology continues to increase with each passing year. It is therefore imperative that training requirements and competencies are routinely reviewed and updated to reflect the changing environment in hospitals and clinics across the country. In 2010, the AAPM Work Group on Periodic Review of Medical Physics Residency Training was formed and charged with updating *AAPM Report Number 90*. This work group includes AAPM members with extensive experience in clinical, professional, and educational aspects of medical physics. The resulting report, *AAPM Report Number 249*, concentrates on the clinical and professional knowledge needed to function independently as a practicing medical physicist in the areas of radiation oncology, imaging, and nuclear medicine, and constitutes a revision to *AAPM Report Number 90*. This manuscript presents an executive summary of *AAPM Report Number 249*.

PACS number: 87.10.‐e

## I. INTRODUCTION

According to the AAPM, medical physics is a branch of physics that applies the concepts and principles of physics to the diagnosis and treatment of human diseases.^(1)^ Medical physics encompasses four subfields: imaging physics, nuclear medicine physics, radiation oncology physics, and medical health physics. However, most clinical residency training programs focus on one of the first three subspecialties. The objective of a medical physics residency training program is to educate and train medical physicists to a level of competency sufficient for independent, professional practice in their specified subfield of medical physics.^2^, ^3^ To accomplish this goal, adequate organization, facilities, staff, patient resources, and educational environments must be provided.^(2)^

## II. STRUCTURE AND CONDUCT OF MEDICAL PHYSICS RESIDENCY PROGRAMS

### A. Length of training

Following the appropriate didactic training (see Section IV),^(2)^ a clinical training period of at least two years is required. The first goal in these two years should be to provide the trainee with a broad experience in clinical medical physics in the subfield in which the residency program specializes. This provides the foundation for the physicist to manage the broad range of medical physics tasks involved in caring for patients in diagnostic and interventional radiology, nuclear medicine, or radiation oncology.

Next, training should build on this clinical foundation in terms of both the level of responsibility and the depth of coverage of topics such as specification, commissioning and acceptance testing, quality assurance, special procedures, and patient safety measures. After two years of clinical training, residents are expected to have sufficient competence to function independently and safely as medical physicists in a clinical environment. Some residency programs may choose to require more than two years of training, allowing residents time to obtain further supervised experience and/or an opportunity to participate in research/developmental projects.

It is important to note that portions of clinical training may take place at affiliated institutions. This form of training is acceptable, provided the recommendations and requirements described in this document are followed and the residency model is consistent with the *AAPM Task Group 133 Report*.^(4)^

### B. Program director

There must be a single, identified program director who is authorized to oversee the medical physics residency program and is accountable for the operation of the program.^(5)^ The program director:^2^, ^5^

1) must contribute sufficient time to the program to ensure adequate direction;
2) is responsible for program organization and direction, as well as instruction and supervision of physics residents;
3) must arrange for the provision of adequate facilities, teaching staff, and clinical and educational resources;
4) must oversee and ensure the quality of the didactic and clinical education at all sites participating in resident education;
5) must oversee and approve all assigned supervisors or lead mentors for resident education at either the primary facility or affiliated institutions;
6) is responsible for the recruitment and appointment of physics residents and must ensure that the appointed residents meet the eligibility requirements listed in Section IV;
7) is responsible for ensuring that physics residents are making satisfactory progress, and for providing appropriate remedial action should this not be the case; and
8) must ensure physics residents' performance is periodically evaluated, and that documented feedback is provided to residents in a timely manner.

With regard to qualifications, the program director:

1) must be certified in the subfield of medical physics that he or she is overseeing by an appropriate certifying board; and
2) should have at least five years of full‐time experience beyond clinical residency training as a qualified medical physicist practicing in the subfield of medical physics that he or she is overseeing.

### C. Program steering committee

A program steering committee shall be formed to review the status of the residency program, track the progress of individual residents, address specific issues that may arise concerning the program or the residents, and discuss any necessary changes to the program. The committee should meet on a schedule sufficient to attend to program matters, but no less than three times annually. The steering committee should consist of the program director, relevant staff, and faculty members involved in residency training. It is recommended that a physician and a resident representative be involved, at least during select portions of the meeting or select meetings. At a minimum, there should be a pathway for residents' concerns to be expressed to the committee. It may also be helpful to have an institutional administrative representative on the committee in a nonvoting position or as a liaison to the committee. Minutes of all meetings should be maintained.

### D. other program staff and personnel

The program must provide adequate staff for the teaching of medical physics in the program's area of specialty.^(2)^ Teaching staff must be qualified in those areas in which they are assigned to instruct and supervise physics residents, and they must devote the necessary time and effort to the educational program. In addition, staff must maintain a professional and respectful work environment that is conducive to inquiry and scholarship.® A commitment to the physics resident training program on the part of staff is essential to the success of the program. Programs are encouraged to assess the commitment and educational performance of staff during their annual reviews. Staff should be engaged in scholarly activities, such as:

1) participating in regional and national scientific societies;
2) participating in their own continuing education;
3) participating in programs developed to improve teaching and/or mentoring skills;
4) producing scientific publications and presentations; and
5) teaching, mentoring, or supervising trainees.

An adequate staff should include at least two full‐time medical physicists certified by an appropriate certifying board, and a diagnostic or imaging radiologist, radiation oncologist, or nuclear medical physician certified by the American Board of Radiology (ABR) or its equivalent, depending on the subfield of medical physics in which the program specializes. The ratio of full‐time medical physicists participating in medical physics resident training to residents should be at least 1:1. It is recommended that access to training from a radiation biologist be available.

### E. Institutional support

The institution sponsoring the medical physics residency program should provide administrative support in terms of funding and space, in addition to clinical and educational resources.^(2)^ Adequate conference room and audiovisual facilities should be provided. A commitment to long‐term funding of the program is essential.

Each resident should be allotted office space within the department and be provided a personal computer with network connections, an email account, telephone, office supplies, and access to a copying machine and scanner.

The financial support provided, including benefits should be clearly defined for the resident prior to his/her entry into the residency program.

### F. Educational environment

The medical physics residency program should be conducted in an environment that encourages the exchange of knowledge, experience, and ideas.^(2)^ Open pathways of communication between residents, physics staff, physician staff, dosimetrists, technologists, therapists, physician residents, and other staff and trainees in the facility are imperative. The existence of other training programs for physicians, dosimetrists, technologists, and other allied health personnel helps foster a productive educational environment.

### G. Residents' scholarly activities

The training program should include opportunities for residents to participate in scholarly activities. These may include presentations at departmental conferences and journal clubs, teaching opportunities, and research. Although optional, clinical research and development projects should be included as part of the clinical training program, provided they do not detract from the residents' clinical training. Opportunities for residents to participate in the clinical commissioning of new technology introduced into a facility will prepare residents and provide them with the necessary skills to practice independently as clinical medical physicists.

### H. Conferences

Conferences and teaching rounds should provide for progressive resident participation.® Adequate frequency of conferences and attendance by physics residents, staff physicists, physicians, and other staff should be documented. Gatherings available to residents could include intradepartmental clinical conferences, such as staff meetings, patient case conferences, and physics meetings. Other conferences could include radiation safety training sessions, quality assurance (QA) committee meetings, and journal reviews. As a complement to resident education, it is encouraged that the physics residents periodically present at institutional conferences.

### I. Documentation of activities

The program should maintain documentation of residents' attendance at departmental conferences, seminars, journal clubs, or other educational activities. In addition, to monitor residents' progress, a log detailing their clinical and didactic activities should be maintained. Residents should maintain these records, but it is the program director's responsibility to periodically review these documents to ensure that they are kept current and residents are progressing adequately in the program.

### J. Library resources

A sufficient variety of journals, reference books, and other resource materials pertinent to medical physics, basic sciences, diagnostic and interventional radiology, nuclear medicine, and radiation oncology should be provided and be immediately accessible for resident study.^(2)^ Physics residents must also have access to a general medical library and to the educational resources available on the Internet.

## III. MEDICAL PHYSICS EDUCATIONAL COMPETENCIES

Medical physics residency programs must develop a curriculum and competencies for their residents. Written expectations regarding residents' performance, behavior, and training schedule should be provided to residents upon their entry into the residency program. These objectives should be discussed with each resident and periodically monitored through an established evaluation process. Following their evaluation, residents should be provided with written feedback regarding their performance. If their performance is deemed inadequate, a course of action for remediation should be developed in a timely manner.

Specific recommendations for curricula and competencies in imaging, nuclear medicine, and radiation oncology physics are detailed in *AAPM Report Number* 249.^(6)^ In addition to providing training in the specific competencies related to a given subfield of medical physics, it is recommended that residency programs include training in the following areas:

1) ethics and professionalism — for example, AAPM Code of Ethics, *AAPM TG* 159,^(7)^ American Board of Radiology Foundation (ABRF) ethics and professionalism modules;^(8)^
2) professional liability;
3) professional activities and societies — for example, AAPM, Radiological Society of North America (RSNA), ABR, American College of Radiology (ACR), American Society for Radiation Oncology (ASTRO), Canadian College of Physicists in Medicine (CCPM), Canadian Organization of Medical Physicists (COMP), Society of Nuclear Medicine (SNM), Society for Imaging Informatics in Medicine (SIIM), and International Society for Optics and Photonics (SPIE);
4) soft skills such as communication, teamwork, and leadership training;
5) administration skills, including

a. personnel management (staffing models),

b. budgeting, and c. billing;

6) accreditation and regulatory agencies;
7) continuous quality improvement (CQI); and
8) U.S. Food and Drug Administration (*FDA)/Safe Medical Devices Act* of 1990 (SMDA).

Programs should be mindful of the six core competencies specified by the American Board of Medical Specialties (ABMS) and the Accreditation Council for Graduate Medical Education (ACGME), and make efforts to integrate these competencies in their curricula and evaluation process. These competencies include: (1) patient care and procedural skills, (2) medical physics knowledge, (3) practice‐based learning and improvement, (4) interpersonal and communication skills, (5) professionalism, and (6) systems‐based practice.^5^, ^9^, ^10^, ^11^ Below are some examples of how these six competencies may be evaluated in medical physics resident education.^10^, ^12^

*Patient Care and Procedural Skills*. Medical physics residents should provide information that is appropriate, accurate, and relevant to the diagnosing or treating of medical conditions. Residents shall develop patient care and procedural skills by:

- demonstrating an understanding of radiological anatomy and physiology in clinical contexts;
- demonstrating an understanding of the major factors that affect patient care;
- providing physicians with appropriate technical and dosimetric information; and d. improving and maintaining medical image quality on a variety of systems.

*Medical Physics Knowledge*. Medical physics residents should be knowledgeable, scholarly, and committed to lifelong learning. A resident's knowledge of medical physics shall include:

- specification, acceptance testing, and quality assurance of imaging and therapeutic equipment;
- measurement and calculation of radiation exposure and dose;
- demonstrations of investigatory and analytic thinking in solving clinical problems; and d. applications of physics problem‐solving skills to clinical medical physics problems.

*Practice‐based Learning and Improvement*. Medical physics residents should investigate and evaluate patient care practices, and appraise and assimilate scientific evidence and improve patient care practices. The resident shall pursue practice‐based learning and improvement which will include:

- investigating and evaluating patient care practices;
- understanding and applying radiation biology and epidemiology to clinical situations;
- assimilating scientific evidence to improve patient care practices;
- contributing to research and development projects in cooperation with radiologists, nuclear medicine physicians, radiation oncologists, and others;
- analyzing results of testing and recognizing unexpected findings;
- investigating equipment performance and possible performance problems; and g. recognizing and correcting personal errors.

*Interpersonal and Communication Skills*. Medical physics residents should demonstrate effective communication with physicians, technologists, service personnel, and professional associates. The medical physics resident shall develop interpersonal and communication skills by:

- demonstrating clear and concise expression, both orally and in writing;
- independently communicating with clinicians, technologists, and others regarding the physical principles of clinical problems;
- demonstrating effective teaching of medical physics and radiation effects to trainees, technologists, and other health‐care professionals;
- interfacing with agents external to the institution, including equipment service personnel, government regulators, and accreditation agency representatives;
- producing accurate, concise, and grammatically correct written reports; and f. listening effectively.

*Professionalism*. Medical physics residents should carry out all assigned duties, adhere to ethical principles, and show sensitivity to a diverse patient population. Residents shall develop professionalism by:

- demonstrating a commitment to carrying out responsibilities;
- understanding professional issues and participating in the activities of professional societies;
- showing leadership and adhering to ethical principles;
- demonstrating sensitivity to diverse patient populations;
- being responsive to the needs of patients and prioritizing those needs over self‐interest;
- respecting patient privacy and confidentiality; and g. demonstrating a commitment to excellence and ongoing professional development.

*Systems‐based Practice*. Medical physics residents should be aware of the system of health care at their institution and effectively call on system resources to provide optimal care. Residents shall undertake systems‐based practices that may include:

- showing competence in information technology (IT) issues such as electronic media, software licensing, levels of access, and information security;
- understanding policy development procedures and quality management systems at the departmental and institutional levels;
- developing knowledge of aspects of the institution's capital equipment procurement process, such as relevant business plans and tender documents;
- being aware of and responsive to the larger context and system of health‐care provision; and e. partnering with managers and providers to assess, coordinate, and improve health care.

## IV. DIDACTIC KNOWLEDGE REQUIREMENTS

Upon satisfactory completion of a medical physics residency program, the graduate will have the knowledge of medical physics comparable to that of a graduate of a Commission on Accreditation of Medical Physics Educational Programs (CAMPEP) accredited medical physics graduate program. This is accomplished most directly by accepting graduates from accredited medical physics graduate programs into the residency program.^(13)^

Alternatively, graduates of non‐accredited medical physics graduate programs and graduates of physics or related graduate programs may be accepted into a medical physics residency program if they can demonstrate they have successfully completed the undergraduate and graduate didactic prerequisites as mandated by the ABR and AAPM. To demonstrate that the resident has the equivalent of a minor in physics, the resident's undergraduate or graduate transcript should include at least three upper‐division (third‐ or fourth‐year undergraduate) physics courses or their equivalent. In addition, *AAPM Report Number 197S* has defined six core graduate‐level topics that must be covered prior to the candidate's completion of a medical physics residency program:^(14)^

1) radiological physics and dosimetry;
2) radiation protection and radiation safety;
3) fundamentals of imaging in medicine;
4) radiobiology;
5) anatomy and physiology; and
6) radiation therapy physics.

These core topics are equivalent to 18 semester credits of coursework.

The contents of these courses should comply with the recommendations provided in *AAPM Report Number 197*.^(13)^ A resident may take these courses during their residency. The program director must approve any coursework taken by a resident during residency training and ensure that the didactic work does not interfere with the resident's clinical training or responsibilities.

## V. NUMBER OF RESIDENTS

The number of residents in the training program must be commensurate with the program's capacity to offer an adequate educational experience.^(2)^ The maximum number of residents in the training program must not exceed the number of full‐time equivalent staff physicists participating in medical physics training in the training program's field of specialization.

## VI. EVALUATIONS

### A. Resident evaluations

The program director is responsible for continuing evaluation of the program and documentation of the educational progress and performance of each resident.^(2)^ To ensure continuous progress assessment, the resident should meet periodically (e.g., biweekly) with the clinical coordinator or rotation supervisor. Monthly meetings of the resident with the program director are recommended. Proper documentation of these meetings will ensure compliance and continuity of assessment.

The resident must be evaluated upon completion of an area of study or clinical rotation. The resident's performance and progress should be assessed periodically by the program faculty (e.g., via an oral or written examination). The results of these evaluations should be discussed with the resident and be documented.

The program director should document any prior training from another institution that is being used to satisfy the training requirements of the program. A resident's prior training may be a reason to modify the training content of a rotation (e.g., by providing the resident with more advanced training). The duration of a rotation or the total length of the clinical residency may not be modified, however. It is the program director's responsibility to counsel, censure, and, after due process, dismiss residents who fail to demonstrate appropriate industry, competence, responsibility, learning abilities, or ethical behavior.

### B. Staff and program evaluations

The training program should provide a means by which residents can give feedback regarding the performance of their clinical supervisors or rotation mentors. Measures should be taken to ensure the confidentiality of residents comments (e.g., the compilation of aggregate reports). Staff evaluations should be reviewed by the program director as evaluations are submitted. If an area of concern is identified, a plan of remediation should be developed and discussed with the staff members of interest.

Upon completion of the program, the resident should be given an opportunity to evaluate the training program in its entirety. This evaluation should include a discussion of the teaching abilities, commitment to the training program, clinical knowledge, and professionalism of the staff and program director.^(5)^ These program evaluations should be reviewed by the program steering committee; items of concerns should be identified and addressed.

### C. Internal program review

The program steering committee should convene an annual meeting to discuss the training program in its entirety. During this meeting, the committee should review evaluations of residents, staff, and the program. Any issues or concerns should be identified and, if necessary, a plan of improvement should be developed. The program steering committee shall ensure the training program remains in compliance with the program self‐study. Changes to the program made after the writing of the self‐study should be identified and discussed. The training program should maintain documentation of the program steering committee minutes.

## VII. WORKING ENVIRONMENT

### A. Professionalism, personal responsibility, and patient safety

The program director, mentors, and staff must set a positive example of professional behavior and educate residents regarding proper professional behavior in the clinic. The clinical culture must stress personal responsibility for each team member's role in patient care. Residents should be provided with direct instruction regarding the medical physicist's responsibility in patient care, as well as the roles of physicians and other staff.

The institution and program should promote and demonstrate a commitment to patient safety, emphasizing that patient safety is first and foremost amongst their core values. Direct instruction on the role of the medical physicist in the safe use of imaging and therapeutic equipment and procedures is integral to a successful program. Resources regarding patient safety are available through several professional societies and national and international organizations^15^, ^16^, ^17^, ^18^ and should be included in the program curriculum.

### B. Access and use of medical information

Residents should be permitted access to relevant medical records and patient information (e.g., hard copy or electronic charts). If such access is permitted, *Health Insurance Portability and Accountability Act* (HIPAA) compliance training must first be provided. Additionally, the program director or program faculty should have an open discussion with the residents about the proper use of patient and human research data; it should be made clear that collecting patient‐related data for research purposes without institutional review board (IRB) approval is not permissible.

### C. Ethics

A residency program should include instruction on medical ethics. Specific attention should be given to ethical decision‐making in medical physics practice. Guidance for ethical training of medical physicists has been provided in *AAPM Report Number 159*.^(7)^ Additional resources, such as open courseware from major universities and the ABR Foundation Ethics and Professionalism modules,^(19)^ are available online.

### D. Supervision of residents

All clinical duties performed by residents should involve some level of supervision commensurate with the task by staff physicists, physicians, or other qualified staff. Supervision of residents performing clinical duties shall also comply with the statutes of the state/province in which the program resides. These statutes may vary widely between states/provinces. Generally, first‐year residents require closer supervision and oversight for day‐to‐day tasks than do second‐year residents. Second‐year residents should be working toward doing more independent duties. The program steering committee and institution or department physician leadership should approve any independent duties given to residents.

### E. Levels of supervision

**General supervision.** This includes clinical duties that are performed under the overall direction and authorization of a supervisor. Performance of these duties does not require the presence of the supervisor.^20^, ^21^

**Direct supervision.** This includes clinical duties that are performed while a supervisor is available within the immediate vicinity of a resident. The supervisor is not required to be physically present, but he or she is available to provide assistance and direction as needed throughout the performance of the procedure.^20^, ^21^

**Personal supervision.** This includes clinical duties that are performed in the physical presence of a supervisor.^20^, ^21^

## VIII. CONCLUSIONS

This summary represents the recommended design of a residency program based on the collective wisdom and experience of many clinical medical physicists, the majority of whom are residency program directors. The complete report, including the recommended curricula and competencies in imaging, nuclear medicine, and radiation oncology, is detailed in *AAPM Report Number 249*.^(6)^ The goal of a clinical medical physics residency program is to train medical physicists to a level of knowledge and competence that permits them to practice independently in one of the three main branches of medical physics. Beyond residency training, it is expected that any professional medical physicist will require additional practical experience and continuing education to attain all of the knowledge listed in this document and/or to be competent in all activities listed in AAPM Report Number 249.

We strongly believe in a structured, formal training model for clinical residencies, and we strongly encourage the development of residency programs in any facility that meets a set of minimum requirements and has the will to meet the goal of independent practice for its graduates. These minimum requirements can also be satisfied through collaboration agreements between two or more facilities.

## Supporting information

Supplementary Material

Supplementary Material
