# Supplementary material for: Essentials and guidelines for clinical medical physics residency training programs: executive summary of AAPM Report Number 249
Source: J Appl Clin Med Phys. 2014 May 8;15(3):4–13. doi: 10.1120/jacmp.v15i3.4763 (PMC5711071; doi:10.1120/jacmp.v15i3.4763)
Supplement: Supplementary file 1 — Supplementary Material [file ACM2-15-004-s001.doc]

Dear Mr. Halvorsen,

We are happy to provide the revised report entitled *“****Essentials and Guidelines for Clinical Medical Physics Residency Training Programs: Executive Summary of AAPM Report Number 249****.”* We were pleased with the initial favorable reviews and are glad to provide responses to the reviews as outlined below.

Additionally, all authors on the submitted report have had a chance to review revisions and are in agreement with its findings.

The reviewers’ comments are bulleted (bold) with responses below.

**Reviewer A:**

**General Comments:**

- **This is an executive summary of a report prepared by an AAPM Work Group. Presumably the report has been approved through the AAPM system although that is not stated anywhere and it’s not on the AAPM website. That being the case my comments are largely limited to clarity although I have inserted a few on the content.**

The status of the Work Group report was supplied through private communication with the executive and associate editor of JACMP. Yes, the report was reviewed by the AAPM Education and Training Committee and Education Council. Additionally, it was submitted to SDMAPP in an earlier form in January 2013. At the time of the executive summary submission to JACMP, the report was approved by Education Council and was submitted to Medical Physics Publishing for final copy editing. The report was posted on the AAPM website on December 5^th^ of this year.

**Specific Comments:**

- **18               What is the exact status of the report?**

As indicated above, the report was posted on the AAPM website (<http://www.aapm.org/pubs/reports/>) on December 5^th^ of this year.

- **44               All of the extended residency programs I have come across have been extended to include research and not further clinical skills. Perhaps a comment is required.**

The text has been edited to state:

“Some residency programs may choose to require more than two years of training, allowing residents time to obtain further supervised experience *and/or an opportunity to participate in research/development projects*.”

- **66               Presumably Section 1.5 refers to the report itself, reference 6? If so this should be stated.**

Thank you for pointing out this oversight. The correct reference should be to Section IV of this manuscript, which was listed as 1.5 in the Work Group Report. This has been corrected in the manuscript.

- **89               I would like to see an expectation that program staff and personnel make some effort to maintain and upgrade their teaching/mentoring/adult education skills in the same way as they are expected to maintain their clinical skills.**

A line item has been added to the list of scholarly activities for staff in Executive Summary that reads:

“participating in programs developed to improve teaching and/or mentoring skills.” Since the AAPM has already approved Report 249, this edit will not be applied to the full report.

- **89               Institutions should also be encouraged to formalize teaching and training in their annual performance assessments of staff.**

A line item has been added to the list of scholarly activities for staff in Executive Summary that reads:

“Programs are encouraged to assess the commitment and educational performance of staff during their annual reviews.” Since the AAPM has already approved Report 249, this edit will not be applied to the full report.

- **157            A definition of competency should be included.**

The Work Group believes the term competency is standard terminology in medical education. We believe this document goes farther in defining specific competencies than prior versions.

- **176            Canadian Organization of Medical Physicists (COMP).**

This acronym has been corrected in the Executive Summary and in AAPM Report 249.

- **267            The wording of this section is problematic. The sentence starting on this line implies that all this knowledge is acquired during the residency which is not the case.**

The Work Group does not believe this sentence implies this knowledge is acquired solely from the education/training a resident would receive from a residency program. However, based on recent changes to ABR and CAMPEP requirements, at the completion of a residency program, a resident should have the didactic background comparable to a graduate of a medical physics graduate program. As the next sentence indicates, “this is accomplished most directly by accepting graduates from accredited medical physics programs into the residency.” Alternative pathway candidates are addressed in lines 273 – 276.

- **276            There need to be web or other references to ABR and AAPM requirements. CAMPEP requires 4 of the six 197S courses to come from accredited graduate or certificate programs.**

A reference to AAPM Reports 197 and 197S were provided in this section (see line 280 and 290). As an AAPM report, this report provides the recommendations of program directors representing the AAPM, not outside entities. Furthermore, our Work Group had extensive discussions with CAMPEP about their requirements, and references to CAMPEP. CAMPEP is currently applying for accreditation through the Council for Higher Education Accreditation (CHEA), and as such must prove to CHEA that it functions completely independently of its sponsoring organizations. Per the request of CAMPEP, we have specifically not referenced CAMPEP in this section.

- **290            Only two of the courses may be taken during the residency without extending it. This whole Section IV needs to be rewritten to make sure it’s compatible with CAMPEP requirements.**

See the response for the comments for line 276.

- **393            It is stated in the Introduction that “The objective of a medical physics residency is to educate and train medical physicists to a level of competency sufficient for independent professional practice…” At line 393 it is admitted that this is not possible. Or else not all the activities listed are necessary for independent, professional practice. This needs to be thought through. Are there core competencies buried in with a lot of others? If so they should be distinguished. Maybe several years beyond a residency are required for peak performance. If this is what is meant then that should be stated. The start and finish of the document are in conflict.**

The Work Group believes that learning does not end at the end of residency and that many items taught during a residency will only be mastered once done many times after the residency.   To clarify this point, we have made some edits to the conclusions of the executive summary.
