# Supplementary material for: Essentials and guidelines for clinical medical physics residency training programs: executive summary of AAPM Report Number 249
Source: J Appl Clin Med Phys. 2014 May 8;15(3):4–13. doi: 10.1120/jacmp.v15i3.4763 (PMC5711071; doi:10.1120/jacmp.v15i3.4763)
Supplement: Supplementary file 2 — Supplementary Material [file ACM2-15-004-s002.doc]

Essentials and Guidelines for Clinical Medical Physics Residency Training Programs: Executive Summary of AAPM Report Number 249

**Joann I. Prisciandaro^1^, Charles E. Willis^2^, Jay W. Burmeister^3^, Geoffrey D. Clarke^4^, Rupak K. Das^5^, Jacqueline Esthappan^6^, Bruce J. Gerbi^7^, Beth A. Harkness^8^, James A. Patton^9^, Donald J. Peck^8^, Robert J. Pizzutiello Jr.^10^, George A. Sandison^11^, Sharon L. White^12^, Brian D. Wichman^13^, Geoffrey S. Ibbott^2^, and Stefan Both^14^**

**Department, Institution, City, State, Country:**

*University of Michigan,***^1^** *Ann Arbor, Michigan*

*The University of Texas MD Anderson Cancer Center,***^2^** *Houston, Texas*

*Wayne State University,***^3^** *Detroit, Michigan*

*UT Health Sciences Center,***^4^** *San Antonio, Texas*

*University of Wisconsin,^5^ Madison, Wisconsin*

*Washington University,^6^ St. Louis, Missouri*

*University of Minnesota,^7^ Minneapolis, Minnesota*

*Henry Ford Hospital System,^8^ Detroit, Michigan*

*Vanderbilt University,^9^ Nashville, Tennessee*

*Upstate Medical Physics,^10^ Victor, New York*

*University of Washington,^11^ Seattle, Washington*

*University of Alabama,^12^ Birmingham, Alabama*

*Texas Oncology,^13^ Round Rock, Texas*

*University of Pennsylvania,^14^ Philadelphia, Pennsylvania*

Corresponding author:

Joann I. Prisciandaro:
*University of Michigan Hospital and Health Systems,*

*Department of Radiation Oncology*

*1500 East Medical Center Dr.*

*UH B2 C432, SPC 5010*

*Ann Arbor, MI 48109, USA*

*Tel.: (734) 936 4309
Fax: (734) 936 7859*
Email: joannp@med.umich.edu

Submitted: October 22, 2013

Accepted: December 19, 2013

Running title: Executive Summary of AAPM Report No. 249
